# Prevalence of occlusal deviations and orthodontic treatment in the Northern Finland Birth Cohort of 1986

**DOI:** 10.2340/aos.v85.45758

**Published:** 2026-04-15

**Authors:** Teresa Kovanen, Elisa Tervahauta, Ville Vuollo, Anna-Sofia Silvola

**Affiliations:** a Research Unit of Population Health, Faculty of Medicine, University of Oulu, Oulu, Finland; b Medical Research Center Oulu (MRC Oulu), Oulu University Hospital, Oulu, Finland; c The Wellbeing Service Country of North Ostrobothia, Finland; d Faculty of Medicine and Life Sciences, University of Tampere, Tampere, Finland; e Wellbeing Service Country of Pirkanmaa, Finland

**Keywords:** Cohort studies, occlusal deviation, malocclusion, orthodontic treatment, adult population

## Abstract

**Objective:**

To investigate the prevalence of occlusal deviations and received orthodontic treatment in a Finnish adult population, and to assess variation by sex.

**Materials and methods:**

The data (*n* = 1,746) are part of the Northern Finland Birth Cohort 1986 (NFBC1986). A clinical oral examination, including 3D intraoral scanning and self-completed questionnaires was carried out when the subjects were 33–35 years old. Registration of occlusion was done in connection with clinical oral examinations and from 3D dental models.

**Results:**

Over half of the subjects (52.5%) had received orthodontic treatment, and it was more common among females compared to males. Almost half (47.5%) had at least one occlusal deviation, with the most common deviations being increased overbite ≥ 5 mm (26.3%), increased overjet ≥ 5 mm (9.2%), anterior crossbite at least in one tooth (5.0%), and lateral crossbite (4.9%). The prevalence of occlusal deviations was higher among males and subjects with orthodontic treatment history compared to females and the untreated group.

**Conclusions:**

Occlusal deviations and orthodontic treatment history were common, although severe deviations were relatively rare. Occlusal deviations were slightly more prevalent among males and in the treated group, whereas orthodontic treatment history was more common among females.

## Introduction

Deviations from normal occlusion, commonly referred to as malocclusions, are frequent in modern populations, with an increasing demand for orthodontic treatment [1–3]. In earlier studies among European populations, the prevalence of occlusal deviations in adults has varied between 40% to 68% [4–8]. Prevalence differs depending on the definition of deviation from normal occlusion, population, and ethnic background [3, 9]. Furthermore, definitions of normal occlusion vary across studies, with the most common differences concerning the acceptable range of overjet and overbite [1, 4, 6–8, 10].

Globally, reports show that the most common deviations from normal occlusion in permanent dentition are crowding, increased overbite, and increased overjet [10]. Moreover, the most common sagittal molar relationship has been found to be Class I and the rarest Class III, the prevalence varying between populations [3, 5, 10, 11].

Due to the craniofacial growth and mesial drift of the molars, the occlusion and dental arch forms change throughout life and occlusion can deteriorate with ageing [12–15]. In addition, relapse and post-treatment changes, such as mandibular anterior crowding, are common after orthodontic treatment [16–18]. However, there are only a few large cohort-based studies that examine the prevalence of occlusal deviations in adults, while most studies have focused on children or adolescents [10, 19]. Previously, the prevalence of occlusal deviations has been examined in middle-aged Finnish adults from the data of the Northern Finland Birth Cohort 1966 (NFBC1966) [6, 20, 21]. However, since then the prevalence of orthodontic treatment has increased significantly among younger generations.

According to the literature, males have more occlusal deviations than females, who instead show significantly higher previous orthodontic treatment experience [4, 6, 7]. In addition, studies show that a higher prevalence of occlusal deviations is associated with the children of parents who have a lower educational level [22]. Although the goal of orthodontic treatment is usually to achieve normal occlusal relationships, previous cohort studies have found that adults with orthodontic treatment history have the same amount or even more occlusal deviations compared to untreated individuals [6, 23].

Over the last decades, access to orthodontic treatment has increased, which is expected to reduce the prevalence and severity of occlusal deviations at the population level. The aim of this study was to examine the prevalence of deviations from normal occlusion and orthodontic treatment history in the NFBC1986 adult population at the ages of 33–35 years. A further aim was to investigate the associations of occlusal deviations with sex, orthodontic treatment history, and educational level.

## Materials and methods

### Subjects

The data for this study were collected from the NFBC1986 [24], which is an epidemiological and longitudinal research programme carried out at the University of Oulu in Northern Finland. The NFBC project collects information on the frequency and symptoms of diseases, as well as the effects of genetic and environmental factors such as social wellbeing. The objective of the NFBC project is to promote health and wellbeing in the population. The study design and population of the NFBC1986 have been described in a previous publication [25].

The NFBC1986 consists of Finnish participants born in the province of Oulu and Lapland with an expected date of birth between 1 July 1985 and 30 June 1986, a total of 9,479 (9,432 liveborn) children born to 9,362 mothers. Subjects living in the city of Oulu and within 250 km of it (*n* = 5,740) were invited to the clinical examination.

**Figure 1:**
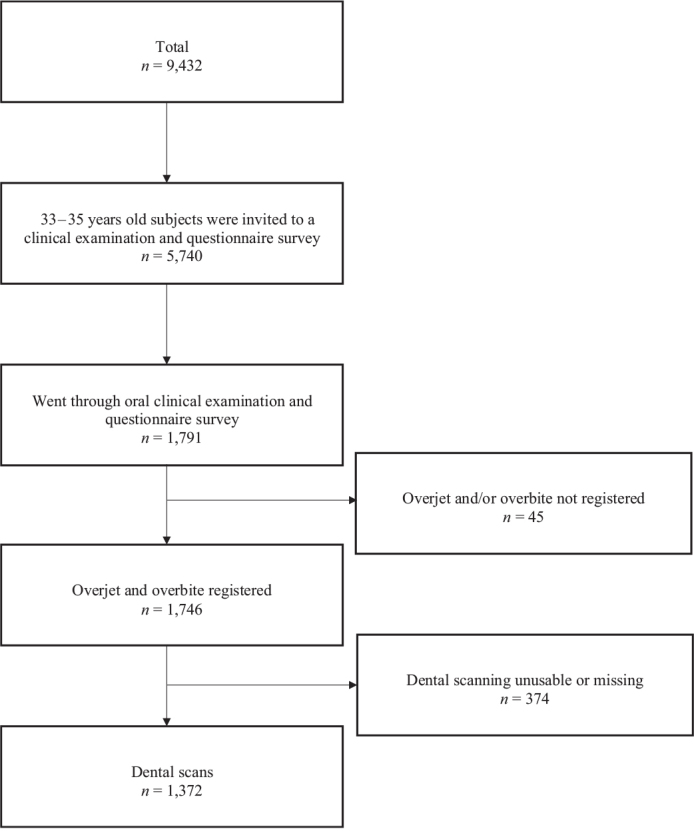
NFBC1986 study population

Of the invited subjects, 1,791 went through an oral examination and questionnaire survey in 2019–2020 when they were between 33 and 35 years old. The oral examination included the registration of overjet and overbite, which were registered from 1,746 subjects (1,047 females and 699 males). Dental scanning was successfully performed on 1,372 subjects. All subjects who had clinically registered overjet and overbite, and/or 3D dental scans, were included in the study. The flow chart of the study population is shown in the Figure 1.

Participation was voluntary and the subjects provided a written consent. The subjects had the right to refuse participation at all phases of the study and were informed about the findings and referred to treatment if needed. The subjects were also given the results of the clinical examination. The investigators were not aware of the identity of the subjects. This study was accepted by the Ethics Committee of the Northern Ostrobothnia Hospital District on 20 February 2019 (108/2017).

### Clinical examination

Extensive oral and dental examinations were performed at the University of Oulu using a standardised clinical dental examinations protocol. Five dentists, who performed examinations, were trained by an experienced specialised dentist and calibrated both before and during the examinations, which increased repeatability of the examination.

Overjet and overbite were measured with a periodontal probe with 2 mm scale in the intercuspal position when there was a maximal number of occlusal contacts, with a precision of 1 mm. Overjet was measured from the labial surface of the right upper central incisor to the most labial surface of the right lower central incisor. Overjet was measured from the left central incisors if the right incisor differed significantly from the dental arch. Overbite was measured between the incisal tips of the right upper and right lower incisors.

### 3D measurements

Dental arches were scanned by using Planmeca Emerald (Planmeca Oy, Helsinki, Finland) and processed with Rapidform2006 software (INUS Technology, Inc., Seoul, South Korea) if needed. Dental scans of inadequate quality were excluded from further analyses. One dentist (T.K.) registered lateral and anterior crossbite, scissor bite, and sagittal molar relationships from those who underwent 3D measurements successfully (*n* = 1,372). The registration was guided by a specialised dentist (A.-S.S.) and calibrated both before and during the examinations. Anterior crossbite, lateral crossbite, and scissor bite were registered according to the registration method developed by Björk et al. [26]. Registration of anterior crossbite included incisors and canines. Registration of lateral crossbite included premolars, first and second molars. Crossbite and scissor bite of third molars were not registered. Sagittal molar relationships were registered bilaterally from the first molars using Angle’s classification [27] and half-cusp-Class II, and further classified into three groups: Class I, Class II, and Class III, the classification having been described previously by Tervahauta et al. [21]. If the first molar was missing, the sagittal molar relationship was not registered from that side. If the sagittal molar relationship was registered unilaterally, that determined general classification.

Intra-examiner agreements for registration of occlusal deviations from 3D scans were assessed. Kappa values for the diagnoses were as follows: sagittal molar relationship right 0.84, sagittal molar relationship left 0.82, crossbite 1.00, and scissor bite 1.00.

### Normal occlusion

In this study, the following definition of ‘normal occlusion’ was used: overjet and overbite of 1–4 mm, no crossbite or scissor bite. Subjects were classified as having ‘deviation from normal occlusion’, herein also termed ‘occlusal deviation’, if there was any deviation according to this definition.

### Questionnaire

Information on orthodontic treatment history, used appliances (fixed/removable), and educational level were gathered in connection with the oral examination using self-completed questionnaires. The questionnaire included the following questions:

‘Have you received orthodontic treatment before the age of 20/after the age of 20?’

‘Was orthodontic treatment performed with fixed appliances/removable appliances/headgear/other appliances or cannot say?’

‘What is your highest education?’ (Dichotomised as 1 = no professional education/vocational or college level education, 2 = polytechnic/university degree)

The data were collected using the anonymous notification channel Webropol. There was also the option of filling in the answers in paper form.

### Statistical analysis

The data of this study were analysed with IBM Statistical Package for the Social Sciences (SPSS) Statistics version 25 using Pearson’s Chi-square test (χ2–test) to calculate the differences between the following groups: females and males; subjects with and without orthodontic treatment history; and educational level 1 and educational level 2. Removable appliances and headgear were combined under the category ‘removable appliances’ for calculation of frequencies. *P–*values less than 0.05 were considered statistically significant.

## Results

The information on orthodontic treatment history is presented in Table 1. Over half of the subjects (52.5%) had received orthodontic treatment either before the age of 20 (51.0%) or in adulthood (4.1%), or both (1.2%). Orthodontic treatment history was more common among females (55.3%) than in males (48.4%) (*p* = 0.004). Subjects with an orthodontic treatment history had significantly more deviations from normal occlusion than untreated subjects (*p* = 0.013).

**Table 1:** Orthodontic treatment history and used appliances among females and males, and treated and untreated subjects.

	Female (%)	Male (%)	Total (%)	*p*
All treated	592 (55.3)	346 (48.4)	938 (52.5)	0.004
Before age 20	573 (53.5)	337 (47.2)	910 (51.0)	0.009
After age 20	57 (5.4)	16 (2.3)	73 (4.1)	0.001
Fixed appliances	204 (35.7)	105 (31.7)	309 (34.3)	
Removable appliances	192 (33.6)	124 (37.5)	316 (35.0)	
Fixed and removable appliances	175 (30.6)	102 (30.8)	277 (30.7)	

Fixed appliances: only fixed appliances used.

Removable appliances: only removable appliances used, included headgear.

Fixed and removable: both fixed and removable appliances used.

Information on orthodontic treatment history available (*n* = 1,785).

Information on used appliances available: female (*n* = 571), male (*n* = 331), total (*n* = 902).

Missing data on used appliances: female (*n* = 21), male (*n* = 15), total (*n* = 36).

In the higher educational group, orthodontic treatment history was significantly more common (57.6%) than in the lower educational group (47.1%) (*p* < 0.001). Subjects with a lower educational level had slightly more (49.8%) occlusal deviations compared to subjects with a higher educational level (45.5%), however, the difference was not statistically significant.

The prevalence of different deviations from normal occlusion traits is presented in Tables 2–4. Of the subjects who successfully went through all the examinations, at least one deviation from normal occlusion was found in 47.5%. The most common occlusal deviations were increased overbite ≥ 5 mm (26.3%), increased overjet ≥ 5 mm (9.2%), anterior crossbite in at least one tooth (5.0%), and lateral crossbite (4.9%). Among males, deviations from normal occlusion were more prevalent than among females (*p* = 0.027).

**Table 2:** Distribution of overjet and overbite among females and males, and treated and untreated subjects.

	Female (%)	Male (%)	Total (%)	*p*	Treated (%)	Untreated (%)	Total (%)	*p*
**Overjet**				< 0.001				0.003
≤ 0 mm	15 (1.4)	35 (5.0)	50 (2.9)		33 (3.6)	16 (2.0)	49 (2.8)	
1–4 mm	938 (89.4)	600 (85.8)	1538 (88.0)		780 (85.4)	741 (90.9)	1521 (88.0)	
5–6 mm	77 (7.3)	51 (7.3)	128 (7.3)		83 (9.1)	43 (5.3)	126 (7.3)	
≥ 7 mm	19 (1.8)	13 (1.9)	32 (1.8)		17 (1.9)	15 (1.8)	32 (1.9)	
**Overbite**				< 0.001				0.02
≤ 0 mm	31 (3.0)	44 (6.3)	75 (4.3)		51 (5.6)	24 (3.0)	75 (4.3)	
1–4 mm	754 (72.0)	457 (65.4)	1211 (69.4)		625 (68.4)	571 (70.3)	1196 (69.3)	
5–6 mm	235 (22.4)	168 (24.0)	403 (23.1)		203 (22.2)	196 (24.1)	399 (23.1)	
≥ 7 mm	27 (2.6)	30 (4.3)	57 (3.3)		35 (3.8)	21 (2.6)	56 (3.2)	

Overjet registered: female (*n* = 1,049), male (*n* = 699), total (*n* = 1,748).

Overbite registered: female (*n* = 1047), male (*n* = 699), total (*n* = 1,746).

Missing data on overbite: female (*n* = 2).

Information on orthodontic treatment history available (*n* = 1,785).

**Table 3:** Distribution of sagittal molar relationships among females and males, and treated and untreated subjects.

	Female (%)	Male (%)	Total (%)	*p*	Treated (%)	Untreated (%)	Total (%)	*p*
Class I	644 (81.5)	442 (80.2)	1086 (81.0)	0.039	562 (78.3)	517 (84.1)	1079 (80.9)	0.012
Class II	137 (17.3)	92 (16.7)	229 (17.1)		137 (19.1)	91 (14.8)	228 (17.1)	
Class III	9 (1.1)	17 (3.1)	26 (1.9)		19 (2.6)	7 (1.1)	26 (2.0)	

Information on sagittal molar relationship available: female (*n* = 790), male (*n* = 551), total (*n* = 1,341).

Information on orthodontic treatment history available (*n* = 1,785).

**Table 4:** Distribution of anterior and lateral crossbite and scissor bite among females and males, and treated and untreated subjects.

	Female (%)	Male (%)	Total (%)	*p*	Treated (%)	Untreated (%)	Total (%)	*p*
Anterior crossbite	35 (4.3)	34 (6.0)	69 (5.0)	0.163	48 (6.6)	21 (3.3)	69 (5.1)	0.006
Crossbite on premolars	32 (4.0)	16 (2.8)	48 (3.5)	0.257	37 (5.1)	11 (1.7)	48 (3.5)	< 0.001
Crossbite on molars	16 (2.0)	22 (3.9)	38 (2.8)	0.034	29 (4.0)	9 (1.4)	38 (2.8)	0.005
Crossbite lateral total	38 (4.7)	29 (5.1)	67 (4.9)	0.728	51 (7.0)	16 (2.5)	67 (4.9)	< 0.001
Scissor bite on premolars	10 (1.2)	2 (0.4)	12 (0.9)	0.083	6 (0.8)	6 (0.9)	12 (0.9)	0.798
Scissor bite on molars	7 (0.9)	10 (1.8)	17 (1.2)	0.137	11 (1.5)	6 (0.9)	17 (1.2)	0.358
Scissor bite total	17 (2.1)	12 (2.1)	29 (2.1)	0.982	17 (2.3)	12 (1.9)	29 (2.1)	0.589

Anterior crossbite included canines and incisors.

Lateral crossbite included premolars, first and second molars.

Subjects with information on crossbite and scissor bite (*n* = 1,372).

Missing data: crossbite on premolars (*n* = 2), crossbite lateral total (*n* = 2), anterior crossbite (*n* = 1).

Information on orthodontic treatment history available (*n* = 1,785).

Most (88.0%) of the subjects had normal (1–4 mm) overjet, while 2.9% of the subjects had overjet ≤ 0 mm, 7.3% had 5–6 mm overjet, and 1.8% had overjet ≥ 7 mm.

More than two-thirds (69.4%) of the subjects had normal (1–4 mm) overbite, while 4.3% of the subjects had ≤ 0 mm overbite, 23.1% had 5–6 mm overbite, and 3.3% had overbite ≥ 7 mm.

Decreased overjet and overbite were more prevalent among males and in the treated group compared to females and the untreated group (*p* < 0.001, *p* < 0.001, *p* = 0.003, *p* = 0.02, respectively). Increased overjet was more common in the treated group than in the untreated group (*p* = 0.003). Males presented more increased overbite than females (*p* < 0.001). Normal overjet and overbite were more prevalent among females and the untreated group than among males and the treated group (*p* < 0.001, *p* < 0.001, *p* = 0.003, *p* = 0.02, respectively).

Class I was the most common sagittal molar relationship (81.0%), while 17.1% had Class II and 1.9% had Class III sagittal molar relationships. Among females and the untreated group, there were more Class I occlusions than males and the untreated group (*p* = 0.039, *p* = 0.012, respectively). The Class II sagittal molar relationship was more prevalent among females and in the treated group (*p* = 0.039, *p* = 0.012, respectively). The Class III molar relationship was more common among males and in the treated group (*p* = 0.039, *p* = 0.012, respectively).

Of all the subjects, 5.0% had anterior crossbite, 4.9% had lateral crossbite, and 2.1% had scissor bite. Males presented more crossbites on molars than females (*p* = 0.034). There was significantly more anterior and lateral crossbites in the treated group than in the untreated group (*p* < 0.05).

## Discussion

The aim of the present study was to examine the prevalence of deviations from normal occlusion and orthodontic treatment history in a Finnish adult population at the ages of 33–35 years, as well as to explore their association with sex. In this study, the most common occlusal deviations were increased overbite and increased overjet, which are globally reported to be among the most common occlusal deviations in permanent dentition [3, 10]. As discussed, earlier studies have shown that subjects with a history of orthodontic treatment and males have a higher prevalence of deviations from normal occlusion compared to untreated subjects and females [4, 6, 7, 20, 23].

The prevalence of orthodontic treatment has significantly increased in recent decades [4, 28–30]. In the previous NFBC1966 study, only 18.6% of the 46-year-old subjects had received orthodontic treatment [6], whereas in the present younger cohort, the prevalence had risen to 53.1%. This finding reflects that orthodontic care for children and adolescents has been widely available in this region for this cohort. In NFBC1966, about half of those with a history of orthodontic treatment received care after the age of 20. In contrast, only a small proportion of subjects in the present cohort underwent treatment in adulthood.

Although the overall prevalence of occlusal deviations in the present study was 47.5%, severe deviations were relatively rare compared to the earlier NFBC1966 study [6]. The criteria for normal occlusion and overjet/overbite deviations were narrower (normal 1–4 mm, increased 5–6 mm, ≥ 7 mm) than in the NFBC1966 study (normal 1–6 mm, increased ≥ 7 mm) [6]. This adjustment was made to better reflect clinically accepted concepts of normal occlusion and to allow better categorisation in a cohort where the prevalence of severe deviations was low and more than half of the subjects had received orthodontic treatment. In a previous study utilising National Health and Nutrition Examination Survey (NHANES) III data, Asiri et al. (2019) defined clinically meaningful occlusal characteristics as an overjet or overbite greater than 4 mm, as well as any reverse overjet or open bite of ≥ 0 mm [7]. Some variation exists in the literature regarding cutoff values for severe deviations, which are typically set at ≥ 6 mm or ≥ 7 mm [4, 7, 8]. Due to the lack of a clear consensus on the limits of malocclusions, the term ‘deviation from normal occlusion’ and ‘occlusal deviations’ was used instead of ‘malocclusion traits’ in this study.

The high prevalence of occlusal deviations in this study aligns with a German study [31]. However, in the German study population, the prevalence of moderate or serious need of orthodontic treatment among adults was even higher (61.6%) when assessed using the Index of Orthodontic Treatment Need (IOTN), which has stricter criteria for accessing orthodontic treatment compared to the present study [31]. Consequently, this indicates a lower need of orthodontic treatment in the present Finnish cohort.

Females have been shown to be more likely to seek and participate in orthodontic treatment compared to males [4, 6, 28, 32]. The more negative impact of occlusal deviations on satisfaction with dental aesthetics among females might partly explain the sex differences in seeking orthodontic treatment [33, 34]. Malocclusions have been found to be more strongly associated with poorer oral health-related quality of life among females [20, 35].

The most common sagittal molar relationship was Class I, followed by Class II, with this finding being in concordance with previous studies [3, 5, 10, 11, 36]. Sagittal molar relationships were not included in the definition of normal occlusion, since adequate occlusion can also be achieved with camouflage treatment through extraction and in cases of hypodontia.

The higher coverage of orthodontic treatment in this cohort compared to the NFBC1966 was reflected in the reduction of individual occlusal deviations. In the present study population, there was less decreased overjet (≤ 0 mm), increased overjet (≥ 7 mm), and increased overbite (≥ 7 mm) among both females and males than in the NFBC1966 study [20]. Interestingly, the prevalence of open bite was at the same level in the cohorts, despite the higher coverage of orthodontic treatment in the younger cohort, which may be due to the difficulty of treating open bite and its high tendency of relapse. In addition, in the NFBC1966 study, subjects were examined at an older age and had a higher prevalence of missing teeth, which may potentially be related to vertical changes.

A systematic review concluded that posterior crossbite in permanent dentition is more common in the European population than in the American, African, and Asian populations [10]. In NFBC1966 study [6], the most common deviation from normal occlusion was lateral crossbite (17.9%), whereas in the present study, only 4.8% of the subjects had lateral crossbite. Furthermore, 2.1% of the subjects had scissor bite, while in the NFBC1966, the number was 7.6% [6]. The differences between the two cohorts could be interpreted to reflect the positive effects of early treatment for transversal problems and its contribution to improved long-term stability.

The present study found that subjects with an orthodontic treatment history exhibited more occlusal deviations compared to the untreated group, whereas in the NFBC1966 study occlusal deviations were at the same level in both the treated and untreated groups [6, 20]. Previous studies have also reported a higher prevalence of certain malocclusions, including crossbite, anterior open bite, and mandibular overjet, among orthodontically treated subjects than among untreated subjects [4, 23]. More severe malocclusions are more likely to receive treatment, and while treatment can reduce deviations, it may not completely eliminate them, particularly in the context of long-term growth and relapse [17]. In addition, orthodontic treatment may have been interrupted, or the outcome may have been compromised due to the patient’s inadequate compliance.

In Finland, publicly funded orthodontic treatment is provided free of charge within the public healthcare system for children and adolescents under 18 years of age, with an emphasis on malocclusions that may cause functional problems [37, 38]. The national guidelines have highlighted the same principles when the cohort participants were children and adolescents [39]. It can be assumed that almost all Finnish children who meet the criteria for admission to treatment receive public orthodontic treatment if the family is willing and the patient is cooperative. In this study, a large part of the treated group had had both fixed and removable appliances. It is also possible that only removable appliances are used in the Finnish public healthcare, with fixed appliances not being necessary, as the primary goal of treatment is to correct the main occlusal problem rather than achieve an ideal occlusion. Due to limited resources, public orthodontic treatment does not include finishing of dental arches unless it is necessary to improve occlusal function. For the above reasons, not all deviations from normal occlusion end up in orthodontic treatment, and some of the subjects have occlusal deviations even though they have received orthodontic treatment.

In the present cohort, orthodontic treatment history was slightly more common in the higher educational level group compared with the lower educational level group. A previous study in adults found that higher education levels were associated with better compliance with orthodontic treatment [40]. Furthermore, previous studies have shown that lower parental socioeconomic status is associated with increased malocclusion and poorer oral health in children [22, 41]. Although parental education was not assessed in this study, the participants’ own educational level likely reflects the socioeconomic environment in which they grew up. Even within Finland’s broad public healthcare system, the small differences detected may stem from greater parental support or better treatment compliance among those in the higher education group.

One strength of this study is a large study population, as well as subjects being born in the limited area in the Northern Finland with an expected date of birth over a period of 1 year. Registration of occlusion was done in connection with a clinical oral examination and from 3D dental models. The examiners were trained by an experienced specialised dentist and calibrated both before and during the examinations.

One limitation of this study was that crowding was not registered. However, crowding is more likely to exist with other occlusal deviations, such as crossbite, Class II and Class III malocclusion [21]. In addition, data collection of orthodontic treatment history would have been more accurate with precise patient files than with self-reported questionnaires. The study did not use a formal orthodontic treatment need index.

## Conclusion

In a Finnish young adult population, the prevalence of deviations from normal occlusion was relatively high (47.5%), although severe deviations were uncommon. A significant portion of the study population had undergone orthodontic treatment (52.5%), reflecting good access to care during growth in this region. This study provides valuable information on the prevalence of occlusal deviations and orthodontic treatment in Finnish adult population, which can be used to assess treatment needs in the future.

## Data Availability

The NFBC data are available from the University of Oulu, Infrastructure for Population Studies. It is possible to apply for permission to use the data for research purposes via the electronic material request portal. The use of data complies with the EU General Data Protection Regulation (679/2016) and the Finnish Data Protection Act. In the most recent follow-up study, the use of personal data is based on written informed consent by the cohort participants, which may cause limitations to its use. Please contact the NFBC project centre (NFBCprojectcenter@oulu.fi) and visit the cohort website (www.oulu.fi/nfbc) for more information.
